# Recent Drugs and Appliances

**Published:** 1887-11-19

**Authors:** 


### RECENT DRUGS AND APPLIANCES.

### Jeyes' Purifiers.

Jeyes' perfect purifiers are well known, and have established for themselves a high reputation. They are made in a variety of portable forms very convenient for use. In addition to the ordinary disinfecting fluid and powder, there are soaps for the toilet, for the laundry, and for the coarser operations of floor scrubbing and scullery washing. Samples of all these varieties of disinfectants have been sent to us, which we have submitted to the usual tests. As might have been expected from the reputation the firm has established, the disinfectant properties of the articles are of the highest efficiency, and well deserve the confidence placed in them by the public. In large towns, not only hospitals, but all kinds of buildings having any drainage system should be freely and frequently disinfected. It is almost impossible by mere flushing to keep ordinary drains sweet and pure. It may be taken as proved without doubt that the abundant use of disinfectants is true economy, in that it is one of the principal factors in checking the spread of fevers and all kinds of contagious diseases.

### Professor Tuson's Disinfectant.

Samples of Professor Tuson's disinfectant have been sent to us. It is prepared both as liquid and powder. Its deodorant properties are very pronounced, destroying the strongest smells almost instantaneously. A little of the liquid used daily for closets and drains keeps the atmosphere quite sweet and wholesome. The powder is very convenient for sprinkling in dustbins, ashpits, and soil receptacles. This disinfectant deserves a place among the most prompt and efficacious articles of its kind.

### Marble Water.

Marble water, as prepared at Margate, is a new candidate for popular favour. It is a plain, purified water, well charged with gas, and is exceedingly palatable. Effervescing waters are very grateful to the palate, and if not indulged in to excess exert a beneficial effect on the stomach and digestion. Sodas, seltzers, and others of the kind containing mineral matter in solution cannot be taken continuously without injury. The marble water, containing nothing but the carbonic acid gas with which it is charged, may be used freely as a substitute for an indefinite period. It is very suitable for mixing with spirits and certain classes of wines.

### Sanitas.

Mr. Justice Kekewich, at the close of his summing up in the action of the "Sanitas" Company against the "Condi-sanitas" Company, said: "To my mind, whether it is 'Condi-sanitas' or 'Sanitant,' it being a compound intended for the same purpose, I must come to the conclusion, and I think any ordinary juryman would come to the conclusion, that he" (the defendant) "has gone as near as he thought he safely could with the intention of cutting out the plaintiffs, and cutting them out dishonestly—that is, passing off his goods as theirs. That is my distinct conclusion on the evidence, and to say that he had put his name, and has not used a yellow label, only goes to show that his impudence was not so great as some fraudulent persons exhibit. I have not the slightest hesitation in granting to the plaintiffs the relief which they claim, and of course with costs."

